# Peroxiredoxins as Markers of Oxidative Stress in IgA Nephropathy, Membranous Nephropathy and Lupus Nephritis

**DOI:** 10.1007/s00005-021-00638-1

**Published:** 2021-12-16

**Authors:** Natalia Krata, Bartosz Foroncewicz, Radosław Zagożdżon, Barbara Moszczuk, Magdalena Zielenkiewicz, Leszek Pączek, Krzysztof Mucha

**Affiliations:** 1 Department of Immunology, Transplantology and Internal Diseases, Medical University of Warsaw, Warsaw, Poland; 2 ProMix Center (ProteogenOmix in Medicine) at the Department of Immunology, Transplantology and Internal Diseases, Medical University of Warsaw, Warsaw, Poland; 3 Department of Clinical Immunology, Medical University of Warsaw, Warsaw, Poland; 4 Institute of Biochemistry and Biophysics, Polish Academy of Sciences, Warsaw, Poland; 5 Faculty of Mathematics, Informatics and Mechanics, University of Warsaw, Warsaw, Poland

**Keywords:** Chronic kidney disease, IgA nephropathy, Lupus nephritis, Membranous nephropathy, Oxidative stress, Peroxiredoxins

## Abstract

IgA nephropathy (IgAN), membranous nephropathy (MN), and lupus nephritis (LN) represent important causes of chronic kidney disease. They belong to the immune-mediated glomerulonephritis (GNs), and have distinct pathogenesis, distinct clinical courses, and variable responses to treatment. Therefore, specific diagnostic procedures are necessary for more effective patient management. Recently, a role for oxidative stress has been proposed in various renal disorders. Thus, molecules related to oxidative stress, such as 2-Cys-peroxiredoxins (PRDXs), may represent plausible candidates for biomarkers in renal pathologies. The aim of this study was to assess whether there are differences between individual GNs and healthy controls in the context of PRDXs serum concentration. We enrolled 108 patients with biopsy-proven IgAN (47), MN (26), LN (35) and 30 healthy age- and sex-matched controls. The serum concentrations of PRDX 1–5 were measured with ELISA assays and correlated with demographic and clinical data. The PRDXs’ concentration varied depending on the GN type. We also observed an association of PRDXs with lower estimated glomerular filtration rates, complement, hemoglobin, and body mass index. Our study indicates that individual PRDX can play roles in pathophysiology of selected GNs and that their serum concentrations may become useful as a new supplementary diagnostic markers in IgAN, MN as well as LN. The results of this study open a new avenue for prospective research on PRDXs in renal diseases.

**Supplementary Information:**

The online version contains supplementary material available at 10.1007/s00005-021-00638-1.

## Introduction

Chronic kidney disease (CKD) is a growing public health problem, affecting approximately 8–13% of the population (Brück et al. 2016; Hill et al. 2016). Furthermore, it is projected that in 2040, CKD will be the fifth leading cause of death in the world (Foreman et al. 2018). Glomerulonephropathies (GNs) such as IgA nephropathy (IgAN), membranous nephropathy (MN), and lupus nephritis (LN) are immune-mediated, although they have different etiologies, and are among the most frequent causes of CKD (Pippias et al. 2017). GNs account for about 20% of CKD cases in most countries, usually affecting young people and carry a lifelong CKD burden (Floege and Amann 2016). IgAN and MN belong to the primary GNs, and their annual incidence is estimated at 2–5 and 1–2 cases per 100,000 adults, respectively (McGrogan et al. 2011). LN develops secondary to systemic disease, and its incidence is estimated at 0.4–0.7 cases per 100,000 population per year (Patel et al. 2006). Unfortunately, GNs frequently progress asymptomatically or present with proteinuria, erythrocyturia or hematuria, edema, and hypertension (Vassalotti et al. 2010). These symptoms are neither specific nor sensitive enough for any GN. The diagnosis is difficult and frequently too late, and still requires histopathological evaluation by kidney biopsy, an invasive procedure with known risks (Mucha et al. 2016). Therefore, alternative, specific, reproducible, and safer methods are needed to facilitate noninvasive diagnosis.

There are specific markers that may help to diagnose glomerular diseases, including galactose-deficient IgA1 and IgG autoantibodies that correlate with IgAN (Placzek et al. 2018), anti-phospholipid 2 receptor antibodies that correlate with the histological picture of MN, and anti-double-stranded DNA antibodies associated with LN activity (Na et al. 2017). Multiple urine and serum proteins (Gao et al. 2018; Krata et al. 2018; Moszczuk et al. 2021; Mucha et al. 2014) or gene polymorphisms (Xie et al. 2020; Pac et al 2021) have been proposed as markers of different kidney diseases in the last decade. However, their diagnostic and/or prognostic utility remains to be validated (Krata et al. 2018; Selvaskandan et al. 2020; Sethi et al. 2019, 2020; Yanagawa et al. 2014). In this study, we focused on oxidative stress-related markers, 2-cysteine peroxiredoxins (2-Cys PRDXs), as potentially discriminatory in renal diseases.

Oxidative stress (OS) is one of the mechanisms involved in the progression of every type of CKD, including GN (Krata et al. 2018). Indeed, specific CKD-related conditions may lead to the overproduction of reactive oxygen species (ROS). It was reported that CKD patients have increased levels of plasma thiol oxidation and carbonylation, but the role of PRDXs in the pathophysiology of kidney diseases remains unknown (Cachofeiro et al. 2008; Krata et al. 2018). PRDXs, which are similar in function to well-known antioxidant enzymes such as catalase and glutathione peroxidase, possess the ability to reduce excessive levels of hydrogen peroxide, one of the major OS mediators (Jeong et al. 2012; Yang and Lee 2015). Importantly, kinetics measurements imply that PRDXs reduce more than 90% of cellular peroxides (Adimora et al. 2010; Perkins et al. 2015; Winterbourn 2008), which predisposes them to being a crucial factor in cellular OS regulation. Oxidative stress has been reported in kidney disease, due to both antioxidant depletions as well as increased production of ROS (Daenen et al. 2019; Irazabal and Torres 2020). The kidney is a highly metabolic organ, rich in oxidation reactions in mitochondria, which makes it vulnerable to damage caused by ROS (Aranda-Rivera et al. 2021). Therefore, OS can accelerate kidney disease progression. Different PRDX isoforms were reported to be involved in diabetic nephropathy (Lee and Lee 2018), ischemia/reperfusion damage (Sharapov et al. 2020), obstructive kidney disease (Hwang et al. 2019), ciliopathies (Zacchia et al. 2020) and acute tubular necrosis (Wu et al. 2017). However, the role of PRDXs in the pathophysiology of glomerular diseases is not well known.

In the current study, we hypothesized that 2-Cys PRDXs could be differentially involved in IgAN, MN, and LN. If so, the PRDX family could serve as additional markers of specific GNs. Therefore, the aim of this study was to evaluate PRDX 1–5 serum concentrations in IgAN, MN, and LN patients and healthy controls.

## Materials and Methods

### Patients

We enrolled 108 patients (GN group) diagnosed by renal biopsy with IgAN (47), MN (26), and LN (35). The exclusion criteria were active infection, current pregnancy, history of malignancy, or previous organ transplantation. The healthy control group was defined by the absence of any kidney disease or other chronic diseases requiring treatment and consisted of 30 age- and sex-matched volunteers. Demographic characteristics of study participants are presented in Table 1. The study was performed in accordance with the Declaration of Helsinki guidelines for research on human subjects and was approved by the Ethics Committee of the Medical University of Warsaw (KB/9/2010 and KB/199/2016), and written informed consent was obtained from all the participants.

**Table 1:** Characteristics of study participants

	Healthy controls	IgAN	MN	LN	GN group	*P* value
Demographics						
Age (years)	44.4 ± (12.9)	43.1 ± (13.3)	54.1 ± (13.4)	45.2 ± (12.4)	46.4 ± (13.6)	0.022
Male (%)**	43.3%	51.1%	57.7%	31.4%	46.3%	0.773
BMI (kg/m^2^)	23.8 ± (3.4)	27.3 ± (3.8)	25.7 ± (4.1)	24.7 ± (7.9)	26.1 ± (5.5)	< 0.001
Laboratory data						
WBC (G/L)	5.6 ± (1.02)	8.2 ± (2.4)	8.6 ± (3.1)	7.0 ± (3.5)	7.9 ± (3.02)	< 0.001
HGB (g/dL)	13.9 ± (1.2)	14.1 ± (1.5)	13.6 ± (1.8)	13.2 ± (1.8)	13.5 ± (2.4)	0.128
HCT (L/L)	0.422 ± (0.036)	0.434 ± (0.042)	0.423 ± (0.052)	0.411 ± (0.056)	0.424 ± (0.049)	0.289
PLT (G/L)	236.2 ± (53.8)	251.3 ± (54.6)	248.5 ± (78.9)	243.6 ± (87.2)	248.1 ± (71.5)	0.842
C3 (mg/dL)*	n.a	114.1 ± (27.7)	106.5 ± (48.9)	94 ± (26.6)	106.1 ± (30.2)	0.006
C4 (mg/dL)*	n.a	26.2 ± (6.9)	29.0 ± (11.3)	16.3 ± (8.0)	22.8 ± (9.0)	< 0.001
Serum creatinine (mg/dL)	0.86 ± (0.14)	1.47 ± (1.11)	1.13 ± (0.46)	1.07 ± (0.68)	1.26 ± (0.87)	0.013
eGFR (ml/min/1.73 m^2^)	92.2 ± (13.7)	71.7 ± (34.4)	74.2 ± (26.2)	82.1 ± (27.9)	75.6 ± (30.5)	0.059
Proteinuria* (g/24 h)	n.a	0.96 ± (1.14)	1.00 ± (1.23)	0.46 ± (0.63)	0.82 ± (1.05)	< 0.001
Total serum protein (g/dL)	7.4 ± (0.3)	7.0 ± (0.6)	6.5 ± (1.2)	7.0 ± (0.5)	6.9 ± (0.7)	0.139
Serum albumin (g/dL)	4.3 ± (0.1)	3.9 ± (0.4)	3.9 ± (0.6)	3.9 ± (0.3)	3.9 ± (0.4)	0.029
Serum α-1 (g/dL)	0.3 ± (0.03)	0.3 ± (0.05)	0.3 ± (0.06)	0.3 ± (0.06)	0.3 ± (0.05)	0.345
Serum α-2 (g/dL)	0.6 ± (0.07)	0.725 ± (0.1)	0.8 ± (0.12)	0.789 ± (0.13)	0.750 ± (0.11)	0.011
Serum β-1 (g/dL)	0.5 ± (0.05)	0.487 ± (0.07)	0.477 ± (0.07)	0.478 ± (0.11)	0.484 ± (0.07)	0.909
Serum β-2 (g/dL)	5.6 ± (1.4)	4.7 ± (2.8)	6.0 ± (1.2)	4.9 ± (2.6)	5.00 ± (2.6)	0.413
Serum γ (g/dL)	1.3 ± (0.2)	1.1 ± (0.2)	0.8 ± (0.3)	1.1 ± (0.3)	1.1 ± (0.3)	0.018

Values are given as mean ± SD. Level of significance was calculated with Chi-squared test and non-parametric Kruskal–Wallis test. *P* < 0.05 indicates that at least one studied group is significantly different from one other group. *Healthy controls group excluded from comparison; **comparison between GN and healthy control; *n.a.* not available; *BMI* body mass index, *WBC* white blood count, *HGB* hemoglobin, *HCT* hematocrit, *PLT* platelets, *eGFR* estimated glomerular filtration rate

### Methods

#### Material Collection

Blood samples were collected once from each of the individuals (fasting) into serum separating tubes (Becton Dickinson, Franklin Lakes, NJ, USA). To obtain serum, blood samples were left to clot at 23–25 °C (room temperature, RT) for 30 min and centrifuged at 2000 RPM at RT. Serum samples were stored in aliquots at − 80 °C until further measurements.

#### PRDX Measurements

The serum concentration of each of PRDX (1–5) was measured using commercially available enzyme-linked immunosorbent assays (EIAab, Wuhan, China). Briefly, the samples and standards were added to the microtiter plate and pre-coated with a biotin-conjugated antibody specific to the target antigen. The standards and samples were added in a determined order the amount of 100 µL per well. Then, avidin-conjugated horseradish peroxidase was added to each microplate well. The enzyme–substrate reaction was terminated by the addition of sulfuric acid solution. Color changes in each well were measured spectrophotometrically at a wavelength of 450 nm on a BioTekPowerWave XS microplate reader (BioTek, Winooski, VT, USA). The targeted antigen concentration was determined by comparing the optical density absorbance of samples to the standard curve. Samples below the detection range for each test were set as the lowest concentration obtained from the standard curve to avoid losing the meaningful part of the results and compute reliable statistical analysis.

#### Biochemical and Clinical Characteristics

Laboratory tests of serum creatinine, proteins, complement, blood morphology, urine analysis, and urinary protein were assayed by routine laboratory techniques. The estimated glomerular filtration rate (eGFR) was calculated according to the Chronic Kidney Disease—Epidemiology Collaboration equation. Body weight in kilograms was divided by the square of height in meters (kg/m^2^) to evaluate body mass index (BMI).

### Statistical Analysis

Statistical analysis was performed in R version 3.6.1. and Statistica 13.1 (StatSoft). Results were expressed as mean ± standard deviation, median ± interquartile range, or a percentage value. All variables were tested for normal distribution by the Shapiro–Wilk test. Non-normally distributed variables were analyzed by non-parametric tests. Comparisons between demographic data were tested by the Kruskal–Wallis test (quantitative variables) and Chi-squared test (qualitative variables), whereas comparisons in biomarker levels between control and GN groups were tested by the Mann–Whitney *U* test. Given that the biomarkers are non-normally distributed, the association between pairs of parameters were analyzed using Spearman’s correlation. To correct for testing multiple hypotheses, in PRDX assessment in three GN groups, we used the Bonferroni method—given a total number of 15 comparative tests: 5 biomarkers and 3 disease groups compared to controls, we considered *P* value < 0.05/15 or 0.0033 statistically significant. Receiver-operating characteristic curves (ROC) were calculated with cutoff points established by binary logistic regression with a significance level of *P* < 0.05 and 95% confidence interval. The ROC analysis results were interpreted as follows: AUC < 0.50, low diagnostic accuracy; AUC in the range of 0.50–0.70, moderate diagnostic accuracy; and AUC > 0.70, high diagnostic accuracy.

## Results

### Discrimination Between GN Patients and Healthy Subjects

**Fig. 1:**
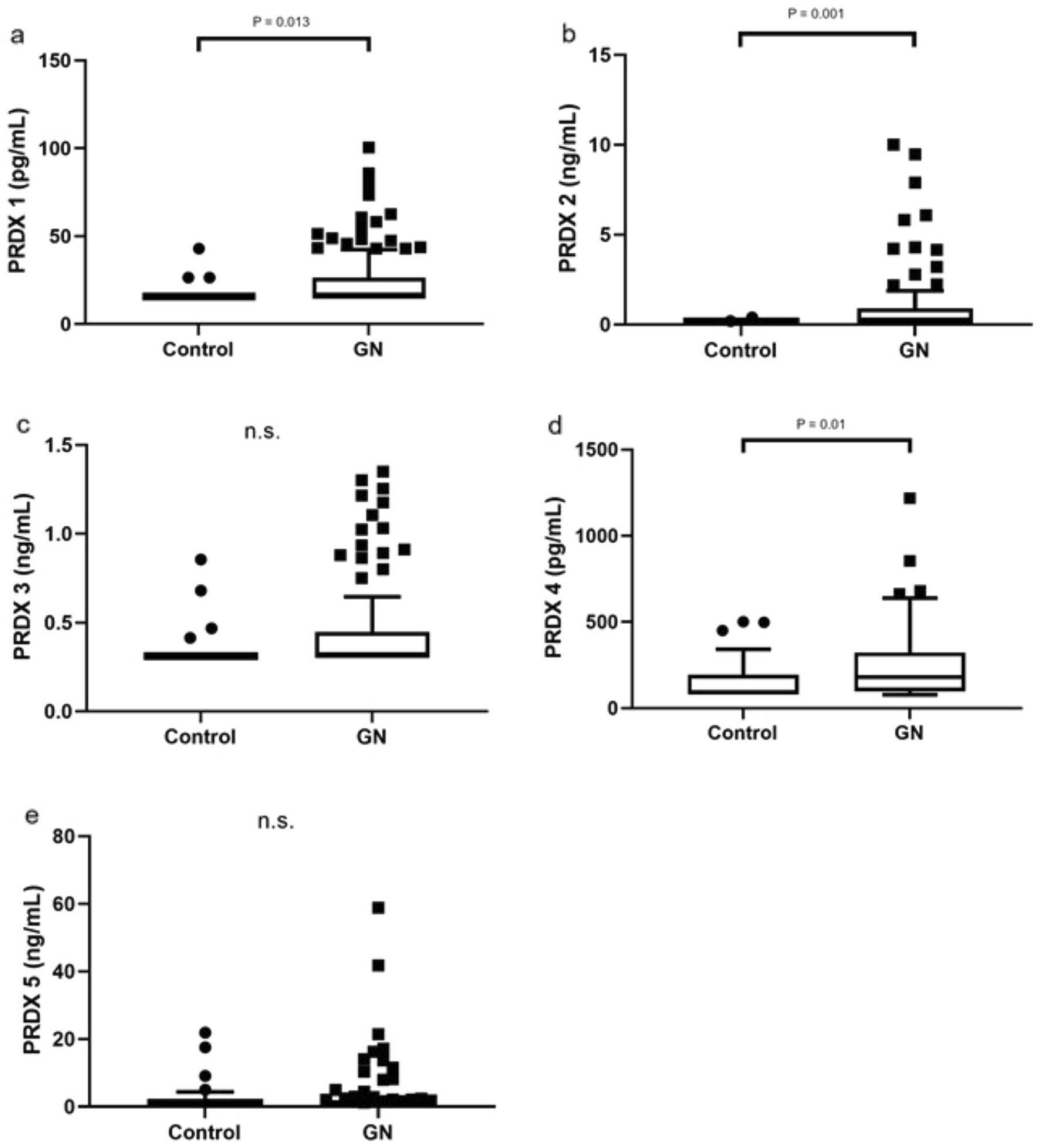
PRDX 1–5 concentrations in patients with GN and healthy controls. Data are presented as box-and-whisker plots; box represents interquartile range (IRQ) with line set as median value for each PRDX concentration, ends of whiskers represent ± 1.5 IQR of value (maximum/minimum), and individual data points indicate outliers. *P* < 0.05 was considered significant (Mann–Whitney *U* test). GN—IgAN, MN, LN combined; (**a**) PRDX 1; (**b**) PRDX 2; (**c**) PRDX 3; (**d**) PRDX 4; (**e**) PRDX 5; n.s.—not significant

We were able to discriminate GN patients in total from healthy subjects based on significantly elevated PRDX 1, 2, and 4 (Fig. 1a, b, d). No differences in PRDX 3 and 5 levels were observed between GN and controls (Fig. 1c, e); however, a clear tendency for higher concentrations of these PRDXs in GN patients has been observed in case of PRDX 3.

### Subclass Variability

**Fig. 2:**
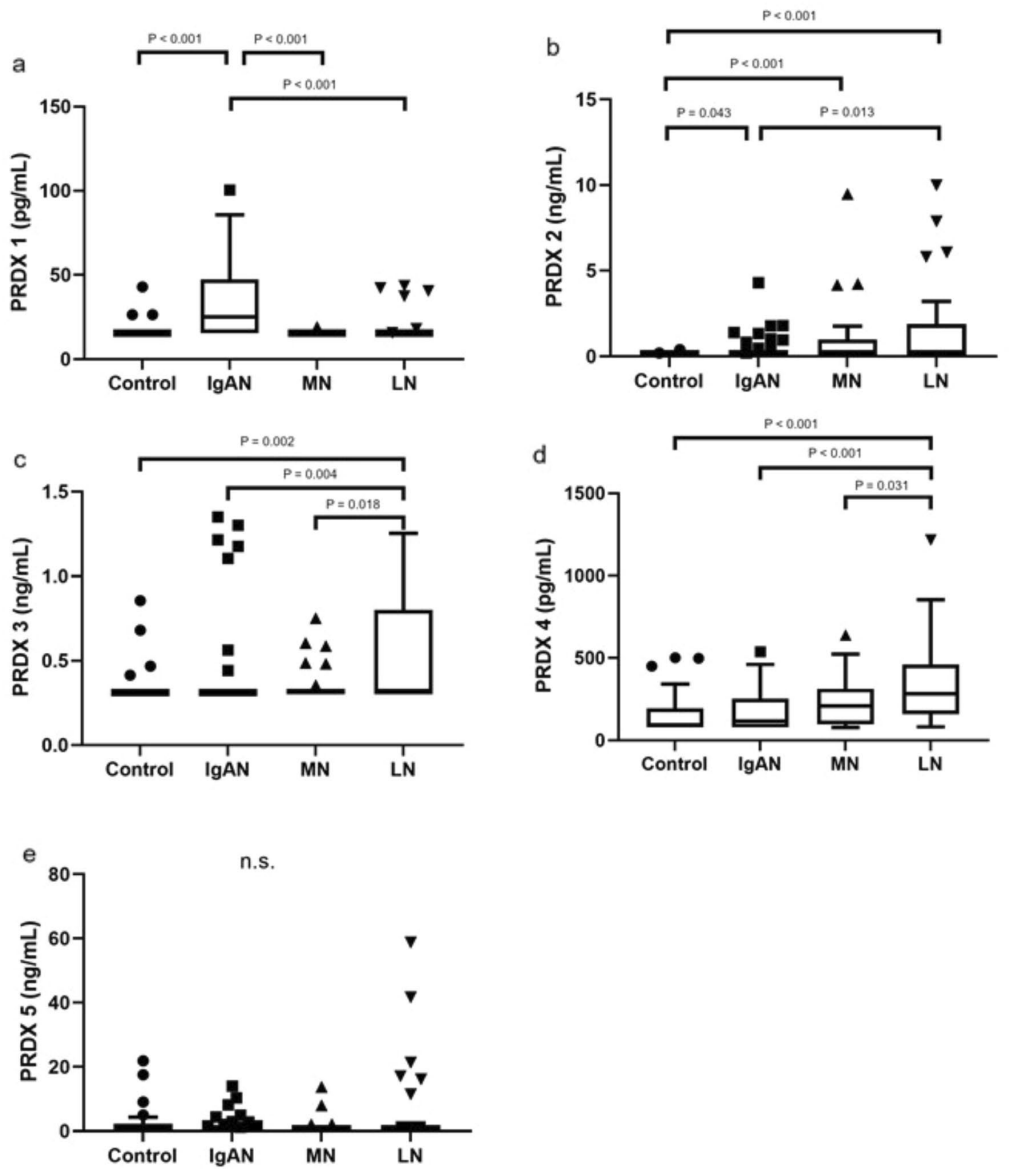
Peroxiredoxin (PRDX) 1–5 concentrations in IgA nephropathy (IgAN), membranous nephropathy (MN), lupus nephritis (LN) and healthy controls. Data are presented as box-and-whisker plots; box represents interquartile range (IRQ) with line set as median value for each PRDX concentration, ends of whiskers represent ± 1.5 IQR of value (maximum/minimum), and individual data points indicate outliers (Mann–Whitney *U* test). *P* value was considered significant if < 0.05 and < 0.033 after Bonferroni correction; (**a**) PRDX 1; (**b**) PRDX 2; (**c**) PRDX 3; (**d**) PRDX 4; (**e**) PRDX 5; *n.s.* not significant

Depending on the PRDX subclass, the serum concentration varied between IgAN, MN, and LN patients and healthy controls (Fig. 2). IgAN patients had significantly higher concentrations of PRDX 1 and 2 (*P* < 0.001 and < 0.043, respectively; however, only PRDX 1 remained significant after Bonferroni correction; Fig. 2a, b); the MN group had almost the same PRDX levels, except for PRDX 2 (*P* = 0.001; Fig. 2b), whereas the levels of PRDX 2, 3, and 4 in LN patients were significantly elevated (*P* < 0.001, < 0.002, and < 0.001, respectively; Fig. 2b–d) compared to those in the control group. Importantly, we noticed significant differences between the disease groups. Comparing IgAN and MN, higher PRDX 1 level was revealed level in IgAN patients (Fig. 2a). The significant differences between IgAN and LN varied depending on the PRDX subclass (1–4) (Fig. 2a–d), whereas comparing LN assessment and MN revealed higher levels of PRDX 3 and 4 in LN individuals (Fig. 2c, d).

To confirm the diagnostic accuracy of PRDX levels, we performed ROC analysis to strengthen the significance of the obtained results. The most prominent AUC values were detected for PRDX 2 (0.652) and 4 (0.653) to discriminate GN from controls and for PRDX 1 to discriminate IgAN from LN (0.733) and MN (0.788) (Table 2).

**Table 2:** Receiver-operating characteristic (ROC) analysis for serum PRDX 1–5 concentration in GN’s and healthy controls

	Area AUC	95% CI (lower)	95% CI (upper)	*P* value	Cutoff point	True positive	False positive	False negative	True negative	Sensitivity	Specificity
PRDX 1											
IgAN_LN	0.733	0.625	0.840	< 0.001	19.107	27	4	20	31	0.574	0.886
IgAN_MN	0.788	0.687	0.890	< 0.001	16.954	28	1	19	25	0.596	0.962
IgAN_Control	0.761	0.656	0.866	< 0.001	16.954	28	3	19	27	0.596	0.900
LN_Control	0.536	0.395	0.677	0.619	15.828	6	3	29	27	0.171	0.900
MN_Control	0.467	0.315	0.619	0.673	19.115	1	3	25	27	0.038	0.900
LN_MN	0.568	0.424	0.711	0.357	15.828	6	1	29	25	0.171	0.962
GN_Control	0.617	0.515	0.720	0.025	15.828	35	3	73	27	0.324	0.900
PRDX 2											
IgAN_LN	0.365	0.240	0.491	0.035	0.165	11	16	36	19	0.234	0.543
IgAN_MN	0.402	0.262	0.541	0.168	0.165	11	11	36	15	0.234	0.577
IgAN_Control	0.590	0.464	0.717	0.163	0.165	11	2	36	28	0.234	0.933
LN_Control	0.710	0.584	0.835	0.001	0.426	15	0	20	30	0.429	1.000
MN_Control	0.688	0.544	0.833	0.010	0.476	9	0	17	30	0.346	1.000
LN_MN	0.543	0.398	0.689	0.560	0.165	16	11	19	15	0.457	0.577
GN_Control	0.652	0.557	0.748	0.002	0.165	38	2	70	28	0.352	0.933
PRDX 3											
IgAN_LN	0.350	0.226	0.473	0.017	0.325	7	17	40	18	0.149	0.514
IgAN_MN	0.470	0.330	0.609	0.670	0.325	7	6	40	20	0.149	0.769
IgAN_Control	0.514	0.382	0.646	0.833	0.325	7	4	40	26	0.149	0.867
LN_Control	0.690	0.561	0.818	0.004	0.513	15	2	20	28	0.429	0.933
MN_Control	0.547	0.394	0.700	0.543	0.480	5	2	21	28	0.192	0.933
LN_MN	0.656	0.521	0.792	0.024	0.513	15	3	20	23	0.429	0.885
GN_Control	0.579	0.472	0.687	0.150	0.325	30	4	78	26	0.278	0.867
PRDX 4											
IgAN_LN	0.230	0.131	0.329	< 0.001	85.899	30	34	17	1	0.638	0.029
IgAN_MN	0.394	0.257	0.531	0.131	79.722	31	20	16	6	0.660	0.231
IgAN_Control	0.545	0.412	0.678	0.507	79.722	31	18	16	12	0.660	0.400
LN_Control	0.800	0.691	0.909	< 0.001	102.148	33	13	2	17	0.943	0.567
MN_Control	0.650	0.504	0.796	0.045	221.942	12	5	14	25	0.462	0.833
LN_MN	0.663	0.522	0.803	0.023	140.977	31	15	4	11	0.886	0.423
GN_Control	0.653	0.543	0.763	0.007	79.722	86	18	22	12	0.796	0.400
PRDX 5											
IgAN_LN	0.472	0.344	0.601	0.674	1.247	11	9	36	26	0.234	0.743
IgAN_MN	0.538	0.400	0.675	0.591	1.247	11	4	36	22	0.234	0.846
IgAN_Control	0.459	0.324	0.593	0.549	1.247	11	9	36	21	0.234	0.700
LN_Control	0.490	0.348	0.631	0.885	1.247	9	9	26	21	0.257	0.700
MN_Control	0.424	0.274	0.575	0.324	1.247	4	9	22	21	0.154	0.700
LN_MN	0.562	0.417	0.706	0.403	1.247	9	4	26	22	0.257	0.846
GN_Control	0.460	0.341	0.580	0.517	1.247	24	9	84	21	0.222	0.700

*P* < 0.05 was considered significant. *AUC* under the curve, *95% CI* 95% confidence interval

### Single-Patient PRDX Concentration Panel (Heatmap)

**Fig. 3:**
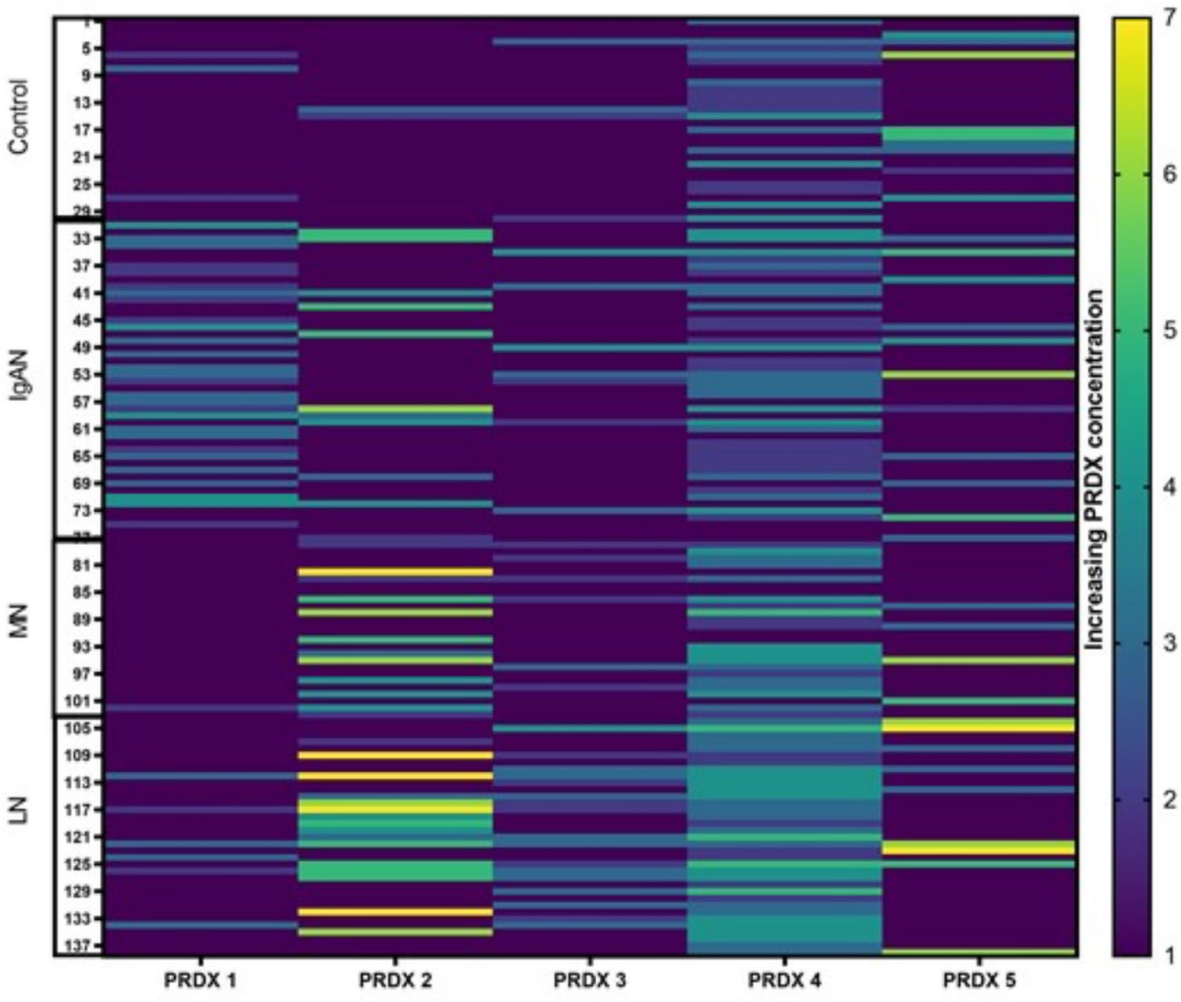
PRDX concentration panel (heatmap) of study participants

Based on our results, we prepared a heatmap (Fig. 3) of PRDX concentrations for all the studied groups. The detection range was set as consecutive concentrations obtained from the standard curve (Table 3). The heatmap suggests the PRDX 1 potential to differentiate IgAN from other GN or controls. Moreover, PRDX 2 concentrations seem significantly higher in LN patients, than in IgAN and controls (as shown in Fig. 2b). This illustrates possible differential GN diagnostic pattern for PRDX 1 and 2; however, to confirm it is applicability, further studies addressing the subclass variability, relationship to such parameters as age, eGFR, proteinuria and anemia are required.

**Table 3:** Consecutive standard PRDX dilutions obtained from standard curve

PRDX	PRDX 1 (pg/mL)	PRDX 2 (ng/mL)	PRDX 3 (ng/mL)	PRDX 4 (pg/mL)	PRDX 5 (ng/mL)
Concentration range					
1	< 15.6	< 0.156	< 0.312	< 78.0	< 0.78
2	15.6–31.2	0.156–0.312	0.312–0.625	78.0–156	0.78–1.56
3	31.3–62.5	0.313–0.625	0.626–1.25	157–312	1.57–3.12
4	62.6–125	0.626–1.25	1.251–2.5	313–625	3.13–6.25
5	125.1–250	1.251–2.5	2.501–5.0	626–1250	6.26–12.5
6	250.1–500	2.501–5.0	5.001–10	1251–2500	12.51–25
7	500.1–1000	5.001–10	10.001–20	2501–5000	25.01–50

### Correlations

#### Glomerular Filtration Rate

**Fig. 4:**
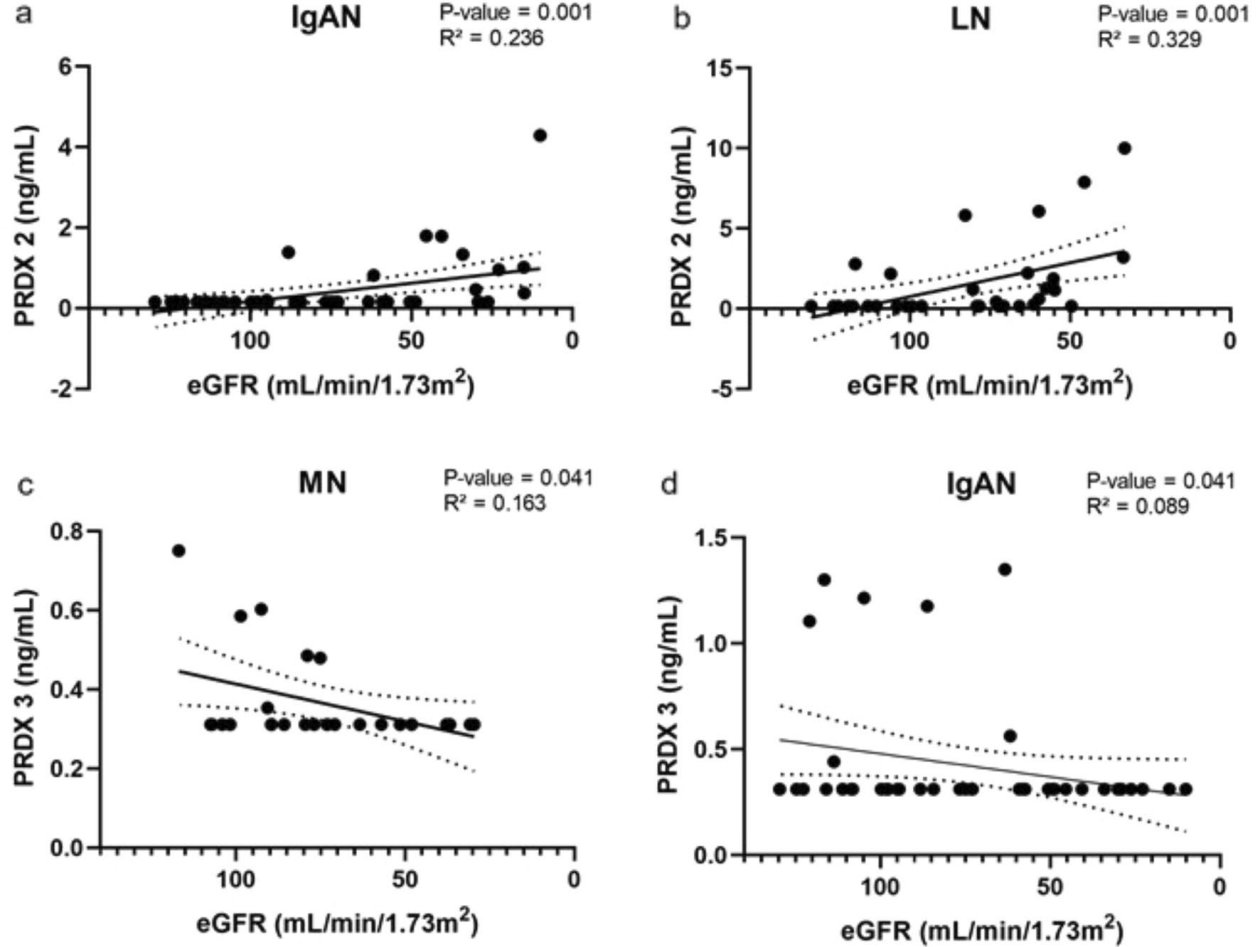
Spearman’s correlation analysis of eGFR and PRDX 2 (IgAN, LN) and PRDX 3 in (IgAN, MN) patients. Correlation was fitted with linear model with 95% confidence interval bands; *P* < 0.05 was considered significant

We observed significant correlation between PRDX 2 serum concentration and renal function as expressed by the eGFR in IgAN (*P* = 0.001) and LN (*P* = 0.001) patients (Fig. 4a, b), and similarly for PRDX 3 in MN (*P* = 0.041) and IgAN (*P* = 0.041) patients (Fig. 4c, d).

#### Complement components C3 and C4

**Fig. 5:**
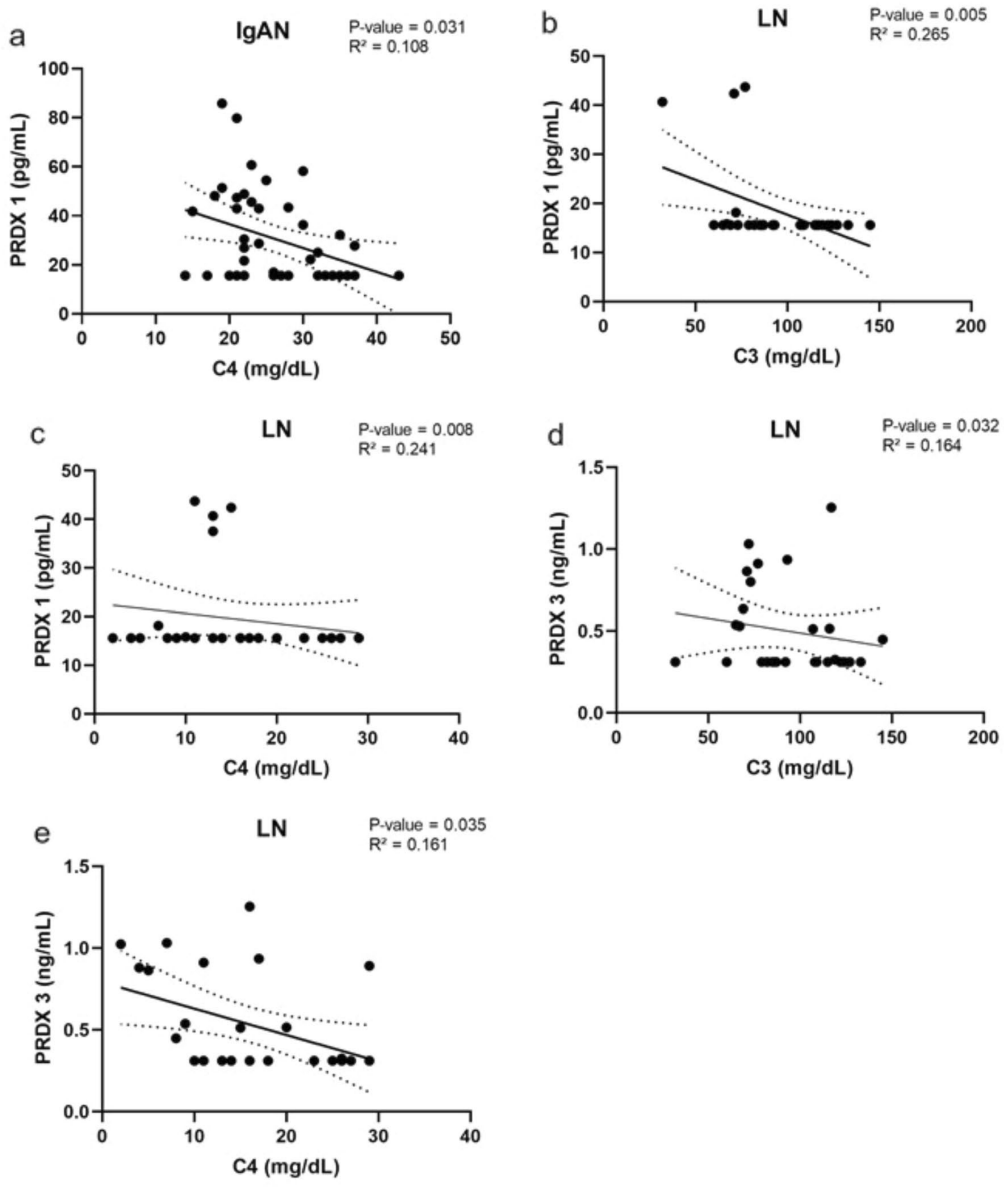
Spearman’s correlation analysis of complements C3 and C4 in IgAN and LN patients. Correlation was fitted with linear model with 95% confidence interval, *P* < 0.05 was considered significant

The complement component concentration showed an inverse association with PRDX 1 in IgAN (*P* = 0.031) and LN (*P* = 0.005 and *P* = 0.008) patients (Fig. 5a, b, c) and PRDX 3 (*P* = 0.032 and *P* = 0.035) in LN patients (Fig. 5d,e). An association was also found with PRDX 3 in the whole GN group (Supplementary Table 1) (Fig. 5).

#### Other Parameters

In addition, a significant inverse correlation between PRDX 2 and hematocrit (HCT) was observed in the GN group (*P* = 0.001). Further analysis revealed that HCT was correlated with PRDX 2 in LN only (*P* = 0.011). The association between hemoglobin (HGB) level and serum PRDX 2 concentration was found in the whole GN group (*P* = 0.001) and in IgAN (*P* = 0.025) and LN (*P* = 0.005) separately (Supplementary Table 1).

We noticed a significant correlation between PRDX 3 and 24 h proteinuria in the whole GN group (*P* = 0.013), but not in IgAN, MN or LN separately. Moreover, 24 h proteinuria was correlated to PRDX 2 only in MN patients (*P* = 0.014) (Supplementary Table 1). At the same time, we did observe an association of PRDX with serum proteinogram changes. For example, in IgAN patients, serum α-1 and β-1 globulins, were correlated significantly with PRDX 1 (*P* = 0.032; *P* = 0.001, respectively), while β-2 globulins were correlated with PRDX 1 (*P* = 0.021), 3 (*P* = 0.018) and 4 (*P* = 0.004). Furthermore, in the MN group, β-2 globulins correlated with PRDX 2 (*P* = 0.033), and in the LN group, β-1 globulins correlated with PRDX 1 (*P* = 0.048) (Supplementary Table 1).

A significant correlation was also found between BMI and PRDX 5 in IgAN patients (*P* = 0.012). Other significant associations are summarized in Supplementary Table 1.

## Discussion

In this study, we show that serum levels of PRDX 1–4 were significantly elevated in IgAN, MN, and LN patients compared to healthy individuals. In addition to the differences between disease and control groups, we also found that each GN type revealed a distinct PRDX pattern. There is no straight explanation why there would be differences in oxidative stress markers between different types of GN. The data on biological reasons for these differences are largely missing.

Oxidative stress is defined as a disturbance in the natural ability of cells to maintain a balance between pro- and antioxidant systems (Krata et al. 2018; Selvaskandan et al. 2020; Sethi et al. 2019, 2020; Yanagawa et al. 2014). Although moderate levels of hydrogen peroxide are necessary for numerous cellular processes, in excessive concentrations it can cause cell and tissue damage (mediated by oxidation of lipids, proteins, or DNA). Several mechanisms are responsible for the removal of reactive oxygen species, including superoxide dismutase, catalase, peroxidase, the peroxide–redox–thioredoxin–thioredoxin reductase enzymatic chain, and a number of non-enzymatic antioxidants (Descamps-Latscha et al. 2001). PRDXs are known as biomarkers for cancer (Basu et al. 2011; O'Leary et al. 2014), bacterial infections (Yang et al. 2018), and neurodegenerative (Goemaere and Knoops 2012) and inflammatory-related diseases (Park et al. 2016). Their role in the pathophysiology and/or diagnostics of IgAN, MN, and LN has not been characterized yet.

The fundamental method of following up CKD, including GN patients, is eGFR estimation. We observed an inverse correlation between eGFR and PRDX 2 in IgAN and LN but not MN patients and PRDX3 in IgAN and MN individuals. For the obvious reasons, a simple link between eGFR and serum PRDX probably does not exist. However, declining eGFR is the evident effect of kidney disease progression involving, e.g., oxidative stress. Coexisting secondary anemia or other comorbidities could additionally influence PRDX levels in serum (discussed below). Another gold standard for GN follow-up is 24-h urine protein loss analysis. Higher proteinuria usually indicates more advanced or active GN. However, we observed only one correlation between PRDX 2 and 24 h proteinuria in MN patients. The implication of this is unclear. The highly variable degree of proteinuria in MN patients, related to the MN biology could explain this finding. On the other hand, a potential relation to protein loss may be suspected also in other GNs based on the observed PRDX association with serum proteinogram changes. Therefore, the causes of proteinogram changes are probably multifactorial and OS is differentially involved. Moreover, to date, there has not been much published data about PRDX and GN, particularly in the context of proteinuria. Of note, proteinuria may additionally serve as an indirect indicator of the GN activity. One could speculate that due to the relative unspecificity of OS, PRDX serum levels might be expected to reflect disease activity rather than disease itself. This speculation could not be elucidated with our study design, since each studied GN has distinct activity scale. Thus, the comparison of disease activity between these groups is impossible. Moreover, diagnostic renal biopsies were performed in history, not at the time of sampling.

OS is implicated in various or almost all disease states, including atherosclerosis, cardiovascular disease, obesity, diabetes, cancer, neurodegeneration, aging, drugs (e.g., allopurinol) and many others. Such conditions could confound the results and anemia belongs to the most potent OS confounders. Along with CKD progression, the uremic toxins increase red blood cell (RBC) damage and erythropoietin deficiency reduces HGB production, resulting in tissue hypoxia. The latter may stimulate OS responses, including, e.g., increased PRDXs (Gwozdzinski et al. 2021). Indeed, we found a correlation between HGB and serum PRDX 2 concentrations in IgAN and LN patients. It was reported that PRDX 2 plays an important role in the protection of RBCs from OS through HGB autoxidation (Johnson et al. 2005), e.g., in iron-deficiency anemia (Nagababu et al. 2008). The protective function of PRDX 2 in HGB stability has been investigated in PRDX 2 knockout mice and RBCs from patients with hereditary hemolytic anemia. The authors of this study suggested that PRDX 2 could bind to HGB and protect it from oxidative denaturation and aggregation in RBCs (Han et al. 2012). What is very important in our study is the fact that our patients had stage 2 CKD and an average eGFR of 71, 74, and 84 mL/min (for IgAN, MN, and LN, respectively), thus had normal RBC and HGB levels. One of the limitations of our study was relatively low patient numbers in each GN group, which precluded analysis of the effect other comorbidities on PRDX concentrations. Therefore, the question is, what does the increased serum PRDX really mean? Do PRDX levels behave as a damage marker or damage type marker? Is it possible that it is just a very early marker of preexisting hypoxia and/or OS in CKD patients long before the development of even early stages of anemia? The fact that different GNs enhance different PRDX classes also suggests different disease-specific mechanisms of hypoxia, which needs additional research. Another question is whether the serum concentrations of PRDXs should be only used as markers for a specific disease type or can also reflects the activity of this particular disease at the time of sample collection? In addition, oxidative stress activity in serum may not always reflect that of changes cellular microdomains in the kidney, which should be taken under consideration while interpreting the results of the current study. Finally, it is also worth adding that evaluation of all GN groups vs. healthy individuals showed significant differences in PRDX 1 and 2 (as illustrated on a heatmap). Therefore, it is an open question whether serum PRDX 1 and 2 might help in the future to distinguish patients suspected of having IgAN, MN-, or LN-related oxidative stress?

Complement activation and its regulation are complex phenomena. It is not definitive whether these phenomena are the direct primary cause or the effector mechanism of kidney injury, but they are involved in GN development and progression; thus, inhibiting them has become an emerging treatment option (Kaartinen et al. 2019). In patients with IgAN, the immunoglobulin A deposited in the mesangial area of the kidney can activate the complement system through either the lectin pathway or an alternative pathway. This may amplify the local inflammatory response and contribute to renal injury (Daha and van Kooten 2016). The immunopathology of human MN shows evidence of complement activation within immune deposits (Beck and Salant 2010). Therefore, it is thought likely that the complement system also plays a substantial role in MN pathogenesis. Although serum levels of complement proteins are usually normal in MN patients, it was recently reported that measuring the circulating complement activation products may be a way to detect ongoing complement activation (Zhang et al. 2019). Complement also has an important role in the pathogenesis of LN. On the one hand, a deficiency of complement components predisposes to lupus, while on the other hand, excess complement activation increases renal damage, and measuring it is done to assess disease activity (Sharma et al. 2020). Although OS involvement in renal injury being driven by complement activation seems obvious, to the best of our knowledge, to date there are no available data linking complement activation in GNs to PRDXs. We assume that the disease-specific PRDX associations with circulating C3 and C4 complement components observed in our study suggest differential OS responses depending on the GN etiology and the mode of complement activation. If so, PRDX subclass assessment might be useful as an adjunct in the diagnosis of GNs.

Another finding of our study is the positive correlation between PRDX 5 and BMI in the IgAN group. It was previously demonstrated in obese mouse models that PRDX 5 inhibits adipogenesis by modulating ROS generation and adipogenic gene expression, implying that it may serve as a potential target to prevent and treat obesity (Kim et al. 2018). Moreover, in vitro and in vivo experiments suggested that PRDX 5 functions as a protective regulator in fatty liver disease and may be a valuable therapeutic target for the management of obesity-related metabolic diseases (Kim et al. 2020). Taking these findings and our results together, it is possible that PRDX 5 is upregulated in response to chronic nephritis depending on BMI value, but this hypothesis needs further elucidation.

It is important to mention that in the current study the serum levels of PRDXs were evaluated, which might not reflect the intracellular changes in PRDXs expression within the tissue. As PRDXs can be released as damage-associated molecular pattern markers from injured tissues (He et al. 2019), this subject warrants further investigations.

Generally, our study indicates potential applicability of antioxidant supplementation in renal disease. The potential compounds to be used are, for instance: coenzyme Q10, Vitamins B, C, D, and E, L-carnitine, statins, or *N*-acetylcysteine (Liakopoulos et al. 2019). Indeed, application of coenzyme Q10 has been reported beneficial in the treatment of chronic diseases, including CKD (Gutierrez-Mariscal et al. 2020).

In conclusion, our results highlight the link between PRDXs and GNs (IgAN, MN, and LN). Our study indicates that individual PRDXs can play roles in pathophysiology of selected GNs and that their concentrations in serum may become useful as new supplementary diagnostic markers in IgAN, MN, and LN. Validation studies including other kidney diseases are required to better understand GNs and enable their less invasive diagnosis.

## Supplementary Information

Below is the link to the electronic supplementary material.

Supplementary file1 (PDF 171 KB)

## Data Availability

Data available upon request.
