# Supplementary material for: Peroxiredoxins as Markers of Oxidative Stress in IgA Nephropathy, Membranous Nephropathy and Lupus Nephritis
Source: Arch Immunol Ther Exp (Warsz). 2021 Dec 16;70(1):3. doi: 10.1007/s00005-021-00638-1 (PMC8677691; doi:10.1007/s00005-021-00638-1)
Supplement: Supplementary file 1 — Supplementary file1 (PDF 171 KB) [file 5_2021_638_MOESM1_ESM.pdf]

Supplementary Table 1. Spearman's correlation analysis of PRDXs in relation to biochemical and clinical characteristics.

Group	Parameter	P-value	R	R^2
GN				
PRDX 1	Serum alfa-1 (g/dL)	0.036	-0.274	0.075
PRDX 1	Serum beta-2 (g/dL)	0.014	0.319	0.102
PRDX 2	Age (years)	0.001	0.306	0.093
PRDX 2	HGB (g/dL)	0.001	-0.395	0.156
PRDX 2	HCT (L/L)	0.001	-0.358	0.128
PRDX 2	Serum creatinine (mg/dL)	0.004	0.274	0.075
PRDX 2	eGFR (mL/min/1.73m²)	0.001	-0.402	0.161
PRDX 3	WBC (G/L)	0.007	-0.260	0.068
PRDX 3	C4 (mg/dL)	0.033	-0.243	0.059
PRDX 3	Serum creatinine (mg/dL)	0.001	-0.341	0.116
PRDX 3	eGFR (mL/min/1.73m²)	0.002	0.301	0.091
PRDX 3	Proteinuria (g/24h)	0.013	-0.250	0.063
PRDX 3	Serum beta-2 (g/dL)	0.026	-0.290	0.084
PRDX 4	Serum alfa-1 (g/dL)	0.009	0.335	0.112
PRDX 4	Serum beta-2 (g/dL)	0.020	-0.302	0.091
PRDX 5	Age (years)	0.006	-0.262	0.069
PRDX 5	BMI (kg/m²)	0.009	-0.250	0.063
IgAN				
PRDX 1	C4 (mg/dL)	0.031	-0.329	0.108
PRDX 1	Serum alfa-1 (g/dL)	0.032	-0.348	0.121
PRDX 1	Serum beta-1 (g/dL)	0.001	-0.555	0.308
PRDX 1	Serum beta-2 (g/dL)	0.021	0.373	0.139
PRDX 1	Serum A/G ratio	0.019	0.378	0.144
PRDX 2	HGB (g/dL)	0.025	-0.328	0.107
PRDX 2	Serum creatinine (mg/dL)	0.005	0.403	0.162
PRDX 2	eGFR (mL/min/1.73m²)	0.001	-0.486	0.236
PRDX 3	Serum creatinine (mg/dL)	0.023	-0.332	0.110
PRDX 3	eGFR (mL/min/1.73m²)	0.041	0.299	0.089
PRDX 3	Serum beta-2 (g/dL)	0.018	-0.383	0.147
PRDX 4	Serum beta-2 (g/dL)	0.004	-0.451	0.204
PRDX 5	BMI (kg/m²)	0.012	-0.364	0.132
MN				
PRDX 2	WBC (G/L)	0.020	0.453	0.205
PRDX 2	Proteinuria (g/24h)	0.014	0.497	0.247
PRDX 2	Serum beta-2 (g/dL)	0.033	-0.642	0.413
PRDX 3	eGFR (mL/min/1.73m²)	0.041	0.404	0.163
PRDX 5	Age (years)	0.007	-0.517	0.268
LN				
PRDX 1	WBC (G/L)	0.025	-0.377	0.142
PRDX 1	C3 (mg/dL)	0.005	-0.515	0.265
PRDX 1	C4 (mg/dL)	0.008	-0.491	0.241

PRDX 1	Serum beta-1 (g/dL)	0.048	0.636	0.404
PRDX 1	Serum A/G ratio	0.042	-0.649	0.421
PRDX 2	Age (years)	0.034	0.360	0.130
PRDX 2	HGB (g/dL)	0.005	-0.465	0.216
PRDX 2	HCT (L/L)	0.011	-0.424	0.180
PRDX 2	Serum creatinine (mg/dL)	0.016	0.403	0.162
PRDX 2	eGFR (mL/min/1.73m²)	0.001	-0.573	0.329
PRDX 3	C3 (mg/dL)	0.032	-0.405	0.164
PRDX 3	C4 (mg/dL)	0.035	-0.401	0.161

Only significant associations with P < 0.05 are presented.
